# Biomimetic Assessment of 3D-Printed T-Shape Joints Bio-Inspired by the Stem-Branch Junction in Common Ash (*Fraxinus excelsior* L.) Trees

**DOI:** 10.3390/biomimetics11010015

**Published:** 2025-12-28

**Authors:** Rastislav Lagaňa, Roman Nôta, Zuzana Tončíková, Tomáš Holeček, Nadežda Langová, Jaroslav Ďurkovič

**Affiliations:** 1 Department of Wood Science, Technical University in Zvolen, 960 01 Zvolen, Slovakia; 2 Department of Furniture and Interior Design, Technical University in Zvolen, 960 01 Zvolen, Slovakia; nota@tuzvo.sk (R.N.); zuzana.toncikova@tuzvo.sk (Z.T.); 3 Department of Wood Processing and Biomaterials, Czech University of Life Sciences, 165 00 Prague, Czech Republic; holecekt@fld.czu.cz; 4 Department of Furniture and Wood Products, Technical University in Zvolen, 960 01 Zvolen, Slovakia; langova@tuzvo.sk; 5 Department of Phytology, Technical University in Zvolen, 960 01 Zvolen, Slovakia; jaroslav.durkovic@tuzvo.sk

**Keywords:** stem–branch junction, common ash wood, 3D printing, T-joints, biomimicry, stiffness, finite element modeling

## Abstract

The stem–branch junction in trees demonstrates exceptional structural design. This study examined two key features of the branch junction in common ash (*Fraxinus excelsior* L.) wood: the interlocked area (ILA) formed above a knot and the spatial arrangement of fibers in the junction. Bio-inspired by the microstructural features revealed by micro-computed tomography imaging, we developed 3D-printed models and compared their mechanical performance to standard symmetrical T-joints. We evaluated the models using mechanical tests and finite element modeling (FEM). Asymmetrical 3D-printed joints mimicking vessel and fiber distribution in the stem–branch junction were 2% stiffer in the elastic region than symmetrical joints and showed, on average, 10% lower deflection at failure. While the ILA had minimal effect on elastic stiffness, measured surface strain analysis indicated that it positively influenced the redistribution of shear strain in the junctions. Thanks to the bio-inspired design, the joints were stiffer and can be utilized in multiple design configurations while maintaining the same underlying principle.

## 1. Introduction

An interdisciplinary approach, called biomimicry or bio-inspired design, studies principles and mechanisms from nature and transfers knowledge into design opportunities [1,2]. The stem–branch connection in trees was an inspiration for many applications. Burns et al. [3] discussed the challenges in replicating tree joint design in composites, emphasizing the need for hierarchical material property refinement to fully realize bio-inspired design benefits. They concluded that 50% flange integration in composite T-joints offers the best trade-off between increased energy absorption and manageable damage initiation. The T-joint showed improved damage tolerance, with higher normalized inelastic strain energy and increased load-carrying capacity following damage initiation. This was attributed to the embedded design that mimicked the tree branch structure. Hawasly et al. [4] explored the structural properties and formation of a tree’s stem–branch junction to develop a bio-inspired, wood-only timber frame joint. They aim to replicate the fiber continuities found in tree joints using bent timber plies as moment-resistant-active fibers.

Branch-to-stem cantilever connections perform well because stem–branch junctions distribute strain efficiently through an optimized shape and locally adapted wood properties [5]. This includes interlocking and tortuous grain that increases tensile and compression strength (e.g., hazel junction apex wood can have about twice the radial and tangential tensile strength of adjacent stem wood) [6]. The higher density and microfibril angle improves fracture toughness and flexibility to manage static and dynamic loads while reducing load transfer to the stem [7].

Fiber direction at hardwood stem–branch junctions is critical. Grain continuity and fiber orientation govern mechanical performance, efficient load transfer, and overall structural integrity [8]. High-resolution methods showed significant variations in fiber orientation near these areas, impacting the overall strength and performance of the wood [9]. In a further study [10], they explored the intricate fiber orientations at the stem–branch junction of Norway spruce to understand the mechanical strength of these connections. Using X-ray computed tomography (CT) at various resolutions, the study traced the fiber directions and presented a 3D visualization of the microstructure. It was found that fibers curve around the knot and integrate with the branch or continue up the trunk, explaining the strong mechanical connection between the trunk and the branch. Thus, the fiber direction at the stem–branch junction of hardwoods is important for maintaining structural integrity, optimizing load transfer, and enhancing mechanical properties. Proper alignment and biomechanical adaptations at these junctions significantly influence the overall performance and durability of wood structures.

The interlocking wood grain above the upper part of the stem–branch interface is an underexplored feature that may affect junction performance. Lev-Yadun and Aloni [11] pointed out that the formation of narrow spiral vessels and non-functional circular vessels decreases the water conductivity of this region and suggested no mechanical function. Contrary to their suggestions, Slater et al. [12] emphasized that the interlocking grains play an important mechanical role in the load-bearing capacity of a branch.

Similarly to interlocking structures in wood, mechanical interlocking also occurs in other biological joining interfaces. Rivera et al. identified interdigitated sutures in the elytra of the diabolical ironclad beetle that significantly increase toughness and resistance to failure, and demonstrated their potential for designing biomimetic interlocking joints in engineering applications [13].

Interlocking as a load-transfer and toughening mechanism is not unique to stem–branch junctions. Nacre also exploits platelet interlocking arising from tablet waviness, which can increase stiffness and shift failure from brittle toward more ductile-like responses. Analytical and numerical results indicate that tuning the interlocking angle can yield simultaneous gains in strength and toughness, suggesting transferable geometric design rules for bio-inspired joints [14].

Additive manufacturing, as a rapidly growing field, uses multiple strategies for increasing the load-bearing capacity and mechanical performance of 3D-printed parts. These include using recycled polypropylene from food packaging [15], optimizing printer configuration and process parameters [16], applying post-processing surface coatings to improve surface properties [17], reinforcing polypropylene and polylactic acid with carbon fibers [18], or using ultrasonic vibrations to strengthen interlayer bonding [19]. Additive manufacturing can also directly implement architected interlocking designs [20], as shown by Djumas et al., who produced single-build, nacre-inspired hybrids where topological interlocking of hard blocks with soft interfaces outperformed conventional brick-and-mortar layouts [21].However, there is still an opportunity for a bio-inspired printing approach that creates joints with enhanced mechanical performance.

This study aimed to develop a 3D-printed frame with a T-shaped connection inspired by the stem–branch junctions in common ash, with emphasis on fiber distribution and the interlocking mechanism observed in natural tree microstructures. Following the biomimicry methodology [22], we identified the key mechanisms behind this natural joint and applied these abstracted design principles to create digital models of T-joints. Models were validated through mechanical testing of both biological and artificial designs.

## 2. Materials and Methods

### 2.1. Plant Material

**Figure 1:**
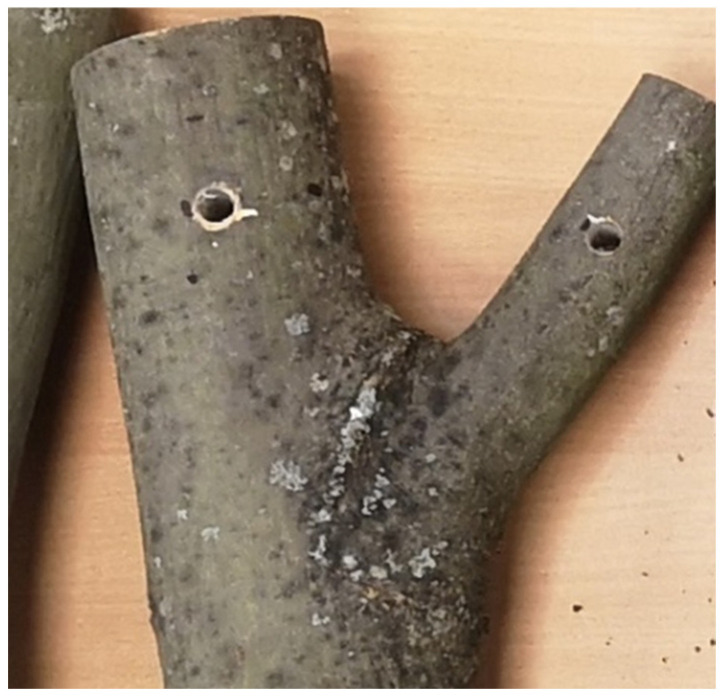
Tree stem–branch junction sample.

The wood material used in this study was sampled from three adult common ash (*Fraxinus excelsior* L.) trees that were harvested in the Arboretum Borová Hora of the Technical University in Zvolen, Slovakia (48°35′ N, 19°08′ E, 337 m a.s.l.). The trees were 45 years old, with a diameter at breast height of 195 ± 35 mm and a branch diameter of 35 mm ± 9.7 mm. Branch sampling was conducted at tree heights ranging from 7 to 10 m. Two sets of samples were prepared for the following experiments:

1. Micro-CT Imaging of the ILA: The first set consisted of 5 × 5 × 20 mm samples prepared from juvenile wood at the expected location of the ILA region near the stem–branch junction. The sample length (20 mm) was oriented along the branch axis and included the first three annual growth rings of the stem–branch junction.

2. Mechanical Testing of Stem–Branch Junctions: The second set included 21 stem–branch junctions obtained from cut trees (Figure 1). Each branch or stem segment was at least 10 cm long. Two parallel holes were drilled into each branch and the upper side of the stem, 50 mm from the junction, to accommodate a loading fixture for mechanical testing. The moisture content of samples was above fiber saturation points.

### 2.2. 3D Printing

Artificial T-shaped samples were 3D-printed from polyethylene terephthalate glycol (PETG) filament (PM PETG; Filament PM, Czech Republic) using a Prusa i3 MK3S+ printer (Prusa Research a.s., Prague, Czech Republic). The perimeters were set to the maximum width avoiding the printing of infills and thus achieving the linear structure of the individual elements of the model. Although the raw PETG material is commonly approximated as isotropic, FDM manufacturing introduces direction-dependent properties due to raster orientation and layer-by-layer deposition and interlayer bonding. Therefore, in this study, the printed specimens were modeled as a linear-elastic orthotropic material with distinct in-plane (XY) and build-direction (Z) properties. To mimic the fiber orientation observed in biological T-shaped connections, the printing direction was designed to reflect this natural pattern. While the biological ILA is spatially oriented in three dimensions, current 3D printing technology restricts the interlocking pattern to the surface of each printed layer. The design and printing pattern of the artificial samples were inspired by two key features: (1) the continuous fiber orientation from “stem” to “branch” and (2) the ILA region above the “stem–branch” interface.

**Figure 2:**
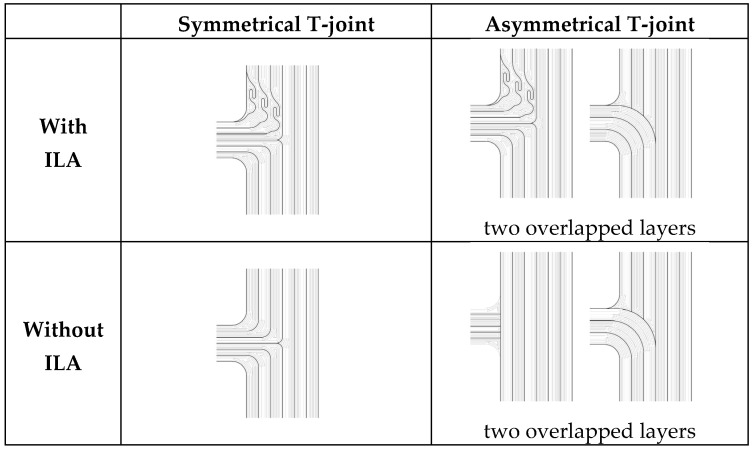
Layering details of 3D-printed samples with or without a feature mimicking the ILA.

Two types of printing patterns for T-joints were created, a common symmetrical alignment (referred to as the “simplified junction model” [4]) and an asymmetrical alignment bio-inspired by stem–branch fiber orientation (Figure 2). All fibers located in a branch have a connection to the root only, and there is no branch–crown fiber flow [10]. The asymmetrical pattern was printed using an alternating two-layer approach to approximate the direction of natural fibers through the 3D printing orientation. Each group of samples was prepared either with or without a lock, mimicking the ILA found in biological structures. A total of 20 replicates were tested for each group.

The samples were printed using a nozzle size of 0.25 mm, a layer height of 0.15 mm, and nozzle speeds of 30 mm/s for the outer perimeter and 20 mm/s for the inner perimeter. The nozzle and heated bed temperatures were set to 240 °C and 90 °C, respectively. The final printed height of each sample was 8 mm.

### 2.3. X-Ray Micro-CT Imaging

Micro-CT scanning of wood samples was performed with a Phoenix V|Tome|X L 240 device (GE Sensing & Inspection Technologies, Wunstorf, Germany) equipped with a 180 kV/15 W high-power nanofocus tube. Scanning parameters were set as follows: voltage 70 kV, current 220 μA, projections 1800, average 3, skip 1, timing 500 ms [23]. Scanning was performed in two phases: an initial full-sample scanning at the voxel resolution of 8 µm, followed by a detailed scanning of the ILA. For the detailed scan, wood material outside the region of interest was removed by sanding, enabling a higher voxel resolution of 2.5 µm. The micro-CT scans were post-processed using the open-source software 3D Slicer [24]. The scans provided detailed images of earlywood and latewood vessel lumens, lumens of libriform fibers, and lumens of parenchyma cells, revealing the spatial distribution of fibers within the ILA.

### 2.4. Mechanical Tests and Data Processing

The ILA region, located above a knot in a standing tree, and the fiber composition, oriented from roots to stem or branch, contribute to variations in the stiffness and strength of the stem–branch junction. These variations depend on whether the load is applied in the direction of branch gravity or the opposite direction. Mechanical tests were performed to assess these stiffness differences and to validate the mechanical behavior of 3D-printed joints by comparing stiffness ratios under both loading directions. A standard method for testing stem–branch junctions does not exist. We used a commonly accepted approach based on the general principles of testing joints with clearly defined boundary conditions, control speed of loading, and reproducible measurements in joint regions [25,26].

**Figure 3:**
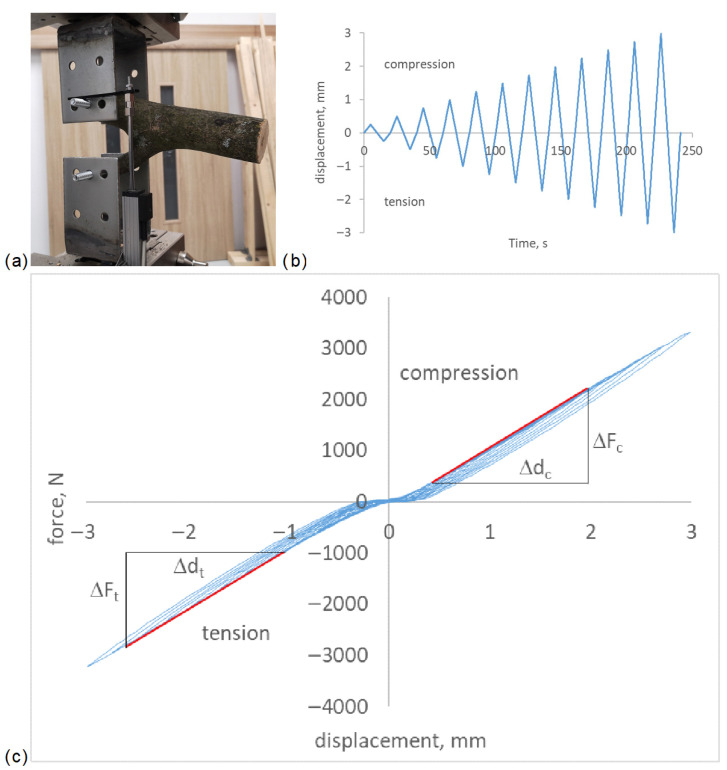
Mechanical evaluation of stem–branch junction stiffness: (**a**) loading setup, (**b**) loading cycle scheme, (**c**) determination of force–displacement stiffness from the linear envelope (red line) of force–displacement curves (blue)

Stem–branch samples were subjected to scissor-like loading using a Labortech 250 testing machine (Figure 3a). The displacement was incrementally increased during tension–compression cycles at a frequency of 0.05 Hz, with a stepwise amplitude increase of 0.25 mm, up to a maximum amplitude of 3 mm in both directions (Figure 3b). The linear portion of the force–displacement envelope, during either compression or tension loading, was used to evaluate the stiffness of the stem–branch junction (Figure 3c).

Stiffness refers to the ability of a material or structure to resist deformation under an applied force. For simplicity, stiffness in compression (*S_c_*) and tension (*S_t_*) can be defined as

(1) $$S_{c/t}=\Delta F_{c/t}/\Delta d_{c/t},$$

where Δ*F_c_* (Δ*F_t_*) is the change in applied force in compression (tension), and Δ*d_c_* (Δ*d_t_*) is the corresponding deformation (Figure 3c). This stiffness definition is dependent on sample size and geometry. To standardize comparisons, a stiffness ratio (*SR*) is introduced, defined as the ratio of stiffness in tension to compression for the same stem–branch sample:

(2) $${SR}_{branch}=S_{t}/S_{c}$$

### 2.5. Mechanical Testing of 3D-Printed Samples

**Figure 4:**
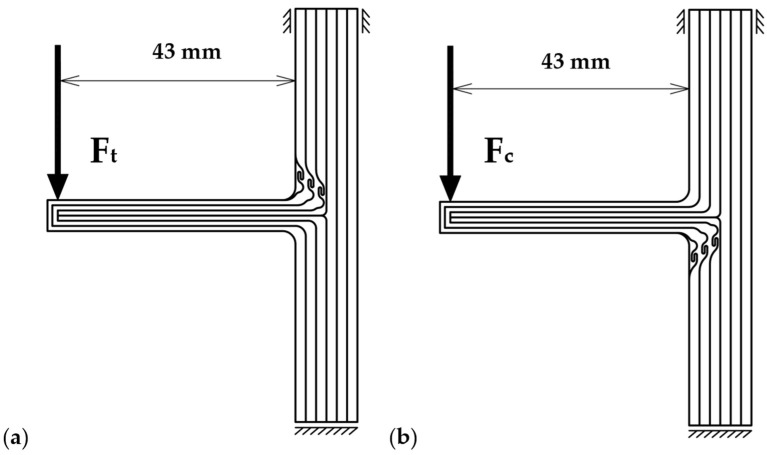
Loading scheme for the 3D-printed samples: (**a**) ‘tensile’ mode, (**b**) ‘compressive’ mode.

A tested 3D-printed sample was secured to the loading stage, and a moment was applied to the “branch” limb. The ILA was located above the limb and was therefore subjected to tension stress under a downward force (Figure 4a). In contrast, the compressive loading of the ILA was achieved by applying the force in the opposite direction (Figure 4b). The loading procedure consisted of a single ramp test conducted at a constant loading speed of 2 mm/min in a temperature-controlled laboratory.

The modulus of elasticity (MOE) in tension and compression mode was calculated according to the following equation:

(3) $$E_{t/c}=F_{t/c}l^{3}/\left(3yI\right),$$

where *F_t_* and *F_c_* are forces creating tension or compression stress in the ILA, *y* is the deflection increment within an elastic part of a force–deflection curve, *l* is the distance of a loading force that created the loading moment in the sample, and *I* is the moment of inertia of a “branch” or side limb, with a cross-sectional area of 6 × 4 mm^2^ (*b* × *h*).

The bending strength *f_m_* of a sample was calculated using the following equation:

(4) $$f_{m}=6F_{t,max}l/\left(bh^{2}\right),$$

where *F_t,max_* is the maximum tension force, and *b* and *h* are the dimensions of the branch cross-section.

The tension/compression ratio (*SR_3D_*) was calculated from the average tension and compression moduli.

(5) $${SR}_{3D}=E_{t}/E_{c}$$

The critical areas of the 3D-printed joints were evaluated by measuring the strain field using the ARAMIS^®^ 3D 12M optical system (GOM, GmbH, Braunschweig, Germany), which operates on the principles of digital image correlation. Due to space resolution, we used larger samples for optical analysis with the cross-section of 12 × 8 mm^2^ loaded at the force distance of 105.5 mm. A random pattern was applied to the sanded surface of the large samples. During loading, the samples were scanned from a distance of 345 mm, with the surface captured at a resolution of 17.3 pixels/mm. The system achieved a subpixel resolution of 0.05 pixels, corresponding to 0.003 mm in physical units. Images were captured at a rate of one frame per minute during the loading process. The strain fields were evaluated and compared at the end of the elastic region at the force of 115 N.

Statistical procedures (analysis of variance and Duncan post hoc test at α = 0.05) were performed using STATISTICA 14.0 (TIBCO Software Inc., San Ramon, CA, USA).

### 2.6. FEM of the Artificial 3D-Printed Samples

Numerical analysis of the 3D model was conducted using the finite element method in ANSYS 2023R1 software. The model utilized 10-node parabolic tetrahedral elements. On average, 319,594 elements and 1,015,308 nodes were used per model. The contact between the printed layers was defined as bonded. In a bonded contact, the two surfaces are constrained such that they neither separate nor slide relative to one another. This implies that the normal and tangential forces are sufficiently high to resist applied forces that would otherwise cause relative motion. Effectively, these forces can approach infinity as the applied load increases.

In the numerical model, PETG was defined as a linear-elastic orthotropic material to reflect the directional anisotropy of FDM structures caused by filament orientation. The calculations were performed using linear static analysis with a linear-elastic orthotropic material model, defined by orthotropic engineering constants *E_XY_*, *E_Z_*, *G_XY_*, *G_XZ_* = *G_YZ_*, *ν* and *ρ*, as specified below. In this context, linear analysis refers to the assumption of a direct proportionality between stress and strain in the material. However, the analysis accounted for geometric nonlinearity (large deformation enabled), understanding that PETG, as a thermoplastic material, undergoes substantial deformation even under normal loading. These deformations can cause significant changes in the model’s geometry during loading, which, in turn, strongly influence the overall behavior of the model and the stress distribution. Therefore, geometric nonlinearity was considered essential and could not be neglected.

In the FEM model, the in-plane modulus was set to *E_XY_* = 1880 MPa, and the build-direction modulus to *E_Z_* = 500 MPa; shear moduli were *G_XY_* = 750 MPa and *G_XZ_* = *G_YZ_* = 300 MPa. Poisson’s ratio was set to *ν* = 0.3, assumed equal for all orthotropic Poisson ratios, and the density was set to *ρ* = 1270 kg/m^3^ [27,28]. A loading force of 16 N was applied, matching the scale of the force within a linear region of the tested sample. To enable comparison with full-field strains measured by the ARAMIS 3D system, larger specimens (cross-section 12 × 8 mm, span 105.5 mm) were also modeled and loaded to 115 N.

## 3. Results and Discussion

### 3.1. Micro-CT Imaging

**Figure 5:**
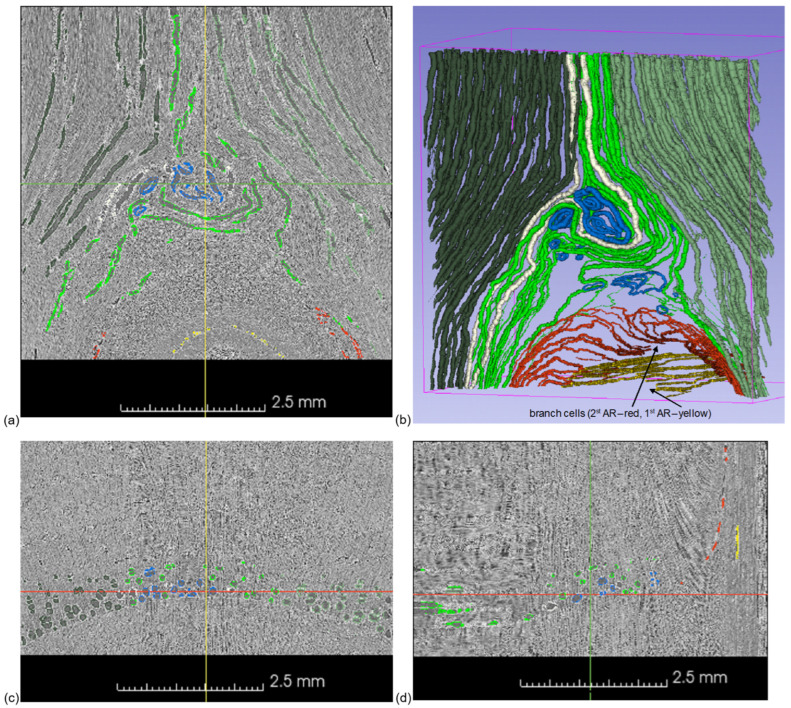
Micro-CT volume of the stem–branch junction, (**a**) cross-section of the branch (red section line in (**c**,**d**), see also Supplementary Video S1), (**b**) 3D view of the stem earlywood and cell lumens of the branch in the 2nd annual ring (AR) and the branch earlywood cell lumens in the 1st AR (3D rotated view can seen in Supplementary Video S2), (**c**) cross-section of the stem (green section line in (**a**,**d**)), (**d**) radial section of the stem (yellow section line in (**a**,**c**)). A color legend of selected earlywood cell lumens: yellow—root–branch lumens of the 1st AR, red—root–branch lumens of the 2nd AR, dark green—right root–crown lumens of the 2nd AR, green—left root–crown lumens of the 2nd AR, light green—root–crown lumens of the 2nd AR twisted around the ILA, blue—ring-like lumens of the 2nd AR in the ILA, pale cream—selected two neighboring root–crown lumens of the 2nd AR twisted around the ILA.

Micro-CT analysis confirmed the presence of two distinct fiber groups that form the stem–branch connections in trees. The first group, referred to as root–branch fibers, extends from the roots to the branch. These fibers either progress directly into the branch volume from the sides or bottom of the trunk or flow around the sides of a knot, gradually turning 90 degrees to “land” on the upper part of the branch (Figure 5).

The second group, known as root–crown fibers, originates in the roots and extends around the knot into the ILA before continuing upward into the crown. The ILA enables these fibers to change direction and intertwine with fibers coming from the right and left sides of the knot, creating a complex and robust structure (Figure 5b). A rotated 3D view of Figure 5b is provided in Video S1. Similarly to Norway spruce [10], we observed a ‘watershed’ boundary that split a group of fibers that integrated with a branch and a group of fibers that continued to the crown.

**Figure 6:**
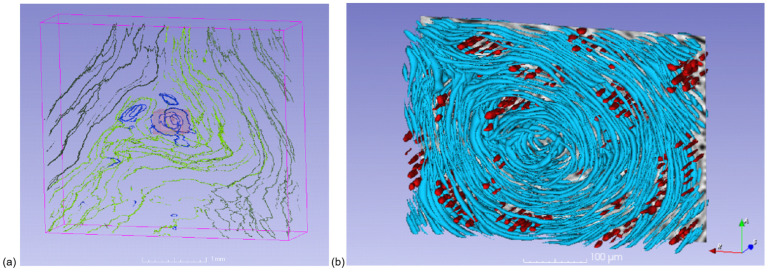
Micro-CT close-up of the ILA within the latewood of the 1st annual ring (AR), (**a**) 3D view of selected latewood cells positioning the detail of the ring-like fiber area. The pale red frame indicates the area of the detail presented in (**b**). (**b**) Detail of the ring-like fiber area (3D rotated view can be seen in Supplementary Video S3). A color legend of selected latewood cell lumens: dark green—right root–crown lumens of the 1st AR, green—left root–crown lumens of the 1st AR, light green—root–crown lumens of the 1st AR twisted around the ILA, blue—ring-like lumens of the 1st AR in the ILA, red—lumens of ray parenchyma cells.

The ILA centers around the root–crown fibers closed ring-like fibers inside which were perpendicularly placed parenchyma cells (Figure 6). The rays were distorted similarly to a fork area in hazel wood [6]. The direction of parenchyma cells was almost parallel to the branch axis. These parenchyma cells give the structure of the ILA additional reinforcement. It was reported that the position and direction of parenchyma cells are in favor of preventing observed tension failure in artificial fiber-reinforced T-joints [3].

### 3.2. Mechanics of Branches

The stem–branch junction stiffness values are presented in Table 1. Stiffness reflects not only the material properties but also the size of the branches. While the stiffness ratio (SR_branch_) is designed to cancel out size effects within a single junction, the observed (SR_branch_) values were unexpectedly negligible and close to 1. This ratio is a far more representative trait due to annulation of the size effect; the standard deviation of the SR_branch_ represents only 3.62% of mean value. A paired *t*-test revealed no significant difference between stiffness values in compression and tension (*p* = 0.2318).

**Figure 7:**
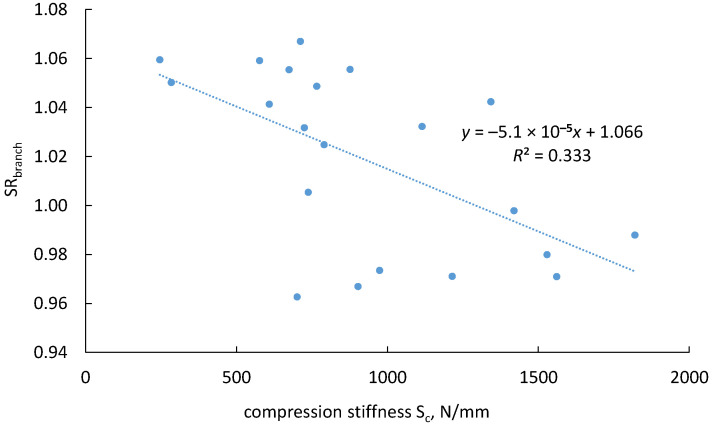
Correlation between tension/compression ratio of the junction stiffness and the compression stiffness.

Further analysis indicated that 33% of the variation in (SR_branch_) could be explained by branch size (Figure 7). A significant correlation was found between the stiffness ratio and compression stiffness: smaller branches exhibited stiffer junctions under tension loading. These findings suggest that the ILA plays a much greater role in enhancing stiffness in small, juvenile branches compared to larger ones.

### 3.3. Mechanical Testing of 3D-Printed Joints

Results showed homogeneity of properties within tested groups. MOE and strength varied from 1.4 to 3.6% and, compared to [29], the variation was very low. Bending strength was on average 5% or 18% higher than bending strength reported by [30,31]. The differences could be attributed to process parameters such as nozzle temperature, feed rate, infill density, and layer thickness [30,32]. The stiffness of the 3D-printed asymmetrical joints resembled that of natural small wood branches (Table 2). For symmetrical joints, the stiffness ratio was 1.01, indicating only a 1% increase in material toughness associated with the presence of ILA. Although this difference was observable, the MOE of the asymmetrical specimens remained essentially unchanged.

**Figure 8:**
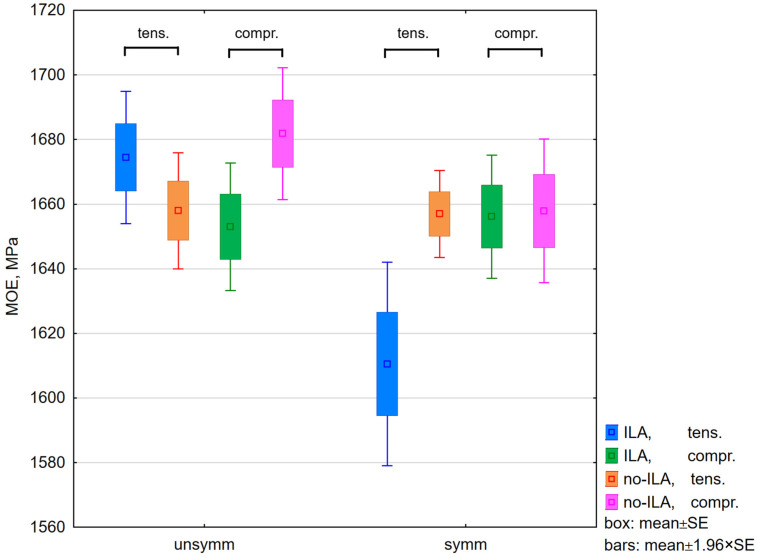
Comparison of stiffness between 3D-printed samples. A box stands for a standard error (SE).

An analysis of variance of the MOE showed that neither ILA nor loading direction was a significant factor (F(1,72) = 3.8, *p* = 0.054; and F(1,72) = 2.5, *p* = 0.118, respectively). No significant interaction between these two factors was detected. In contrast, the MOE was significantly influenced by symmetry alone (F(1,72) = 7.7, *p* = 0.007). In symmetrical joints, the presence of ILA led to a significant reduction in tensile stiffness (Duncan test, *p* = 0.006) (Figure 8). When ILA was assumed to have no effect, the stiffness differences between asymmetrical and symmetrical joints were 2.0% under tensile loading and 0.6% under compression.

The strength response of the 3D-printed joints differed from their stiffness behavior (Table 2). Loading direction was a significant factor (F(1,72) = 11.4, *p* = 0.001), primarily because the asymmetrical joint without ILA showed low strength in the tension mode. Across the full dataset, the presence of ILA was not statistically significant (F(1,72) = 2.72, *p* = 0.108). Nevertheless, when ILA was positioned to mimic its natural configuration in 3D-printed joints (i.e., under tensile loading), joint strength increased slightly (by 2.3%). According to Duncan’s test, this difference was close to significance (*p* = 0.051).

The strain at failure revealed stiffer behavior of a tree-like 3D-printed joint. Both ILA and symmetry significantly affected strain at failure (F(1,72) = 8.82, *p* = 0.004; F(1,72) = 66.75, *p* < 0.001). In tension mode, the asymmetrical joint with ILA showed significantly lower deflection at failure (8% lower) than its counterpart without ILA (Duncan *p* = 0.001). In all cases, the symmetrical layout exhibited on average 10% higher deflection at failure and consequently less stiff behavior. In contrast, elastic deflection at 16 N showed no ILA effect (F(1,72) = 0.0, *p* = 0.832), which confirms that ILA mainly increases the plastic (post-elastic) stiffness of asymmetrical joints in tension.

Failure typically initiated at the neck of the limb. ILA influenced the failure mechanism only indirectly, by redistributing stress. This mirrors real stem–branch junctions, where, under high loads, failure usually occurs in the branch itself rather than at the junction. Such a mechanism minimizes damage to the branch collar, which is essential for wound closure and for protecting the tree against pathogens. This adaptive strategy supports efficient wound coverage and helps maintain overall tree health [33].

When comparing the experimental deflection values *y* (mm) under a load of 16 N (3.09–3.21 mm) with the FEM simulation results (3.538–3.719 mm) (Table 2), the experimental specimens exhibited approximately 10–15% lower deformation than the numerical model, depending on specimen type and loading mode. The smallest discrepancy occurred for the symmetric specimen without the ILA in compression (9.9%), whereas the largest was observed for the asymmetric specimen without the ILA in tension (15.0%). Nevertheless, the FEM model consistently reproduced the mechanical trend observed experimentally. Specimens with higher stiffness showed lower deflection both in the experiment and in the simulation, and the relative ranking between tension and compression remained preserved.

Real 3D-printed specimens display higher effective bending stiffness because bending induces mutual press-fitting of layers as well as partial polymer strain-hardening, effects that are not captured in the FEM model. The FEM simulation assumes ideal bonding between layers without interlayer shear slip and without friction, which does not correspond to the actual bending behavior of FDM specimens. In real 3D bodies, layers are pressed against each other, mechanical interlocking and friction arise at the contacts, and these mechanisms increase bending stiffness. Neglecting these effects leads to an underestimation of stiffness in the FEM model and an overestimation of the calculated deflection compared with the experiment [27,34]. A deformation difference of 10–15% is even smaller than deviations reported in the literature, where FDM polymers often show discrepancies on the order of tens of percent between numerical simulations and experimental behavior due to material homogenization and simplified modeling of interlayer interfaces [28,35]. The FEM model used here can therefore be considered a reliable and conservative tool for comparative structural analysis of the influence of layer orientation and the presence of the ILA in T-joints.

**Figure 9:**
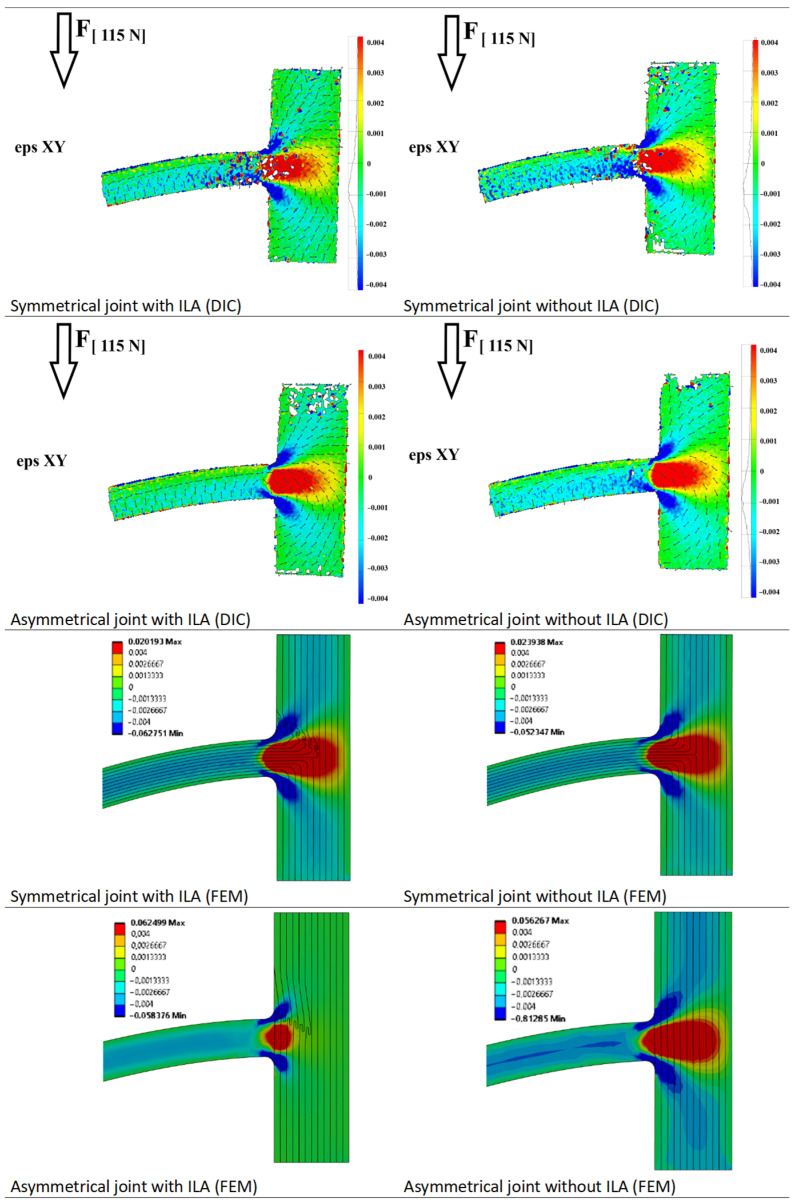
Shear strain distribution of large tested samples and large modeled samples loaded in ‘tension mode’ at 115 N. Arrows represent the major strain direction.

The measured strain field of large tested samples loaded at 115 N pointed to possible reasons for ILA mechanical performance (Figure 9). Since the shear strain showed to be a liability of the artificial T-joints [36], we focused on this feature. A larger shear strain field in the ILA region observed in asymmetric joints pointed to the ILA capability of better shear stress energy distribution. The ILA demonstrated the reduction of shear strain in the lower part of a bent branch in both asymmetric and symmetric joints. The ILA located above the branch redistributed shear strain energy more effectively, thus lowering the effect of shear stress on total deflection. The normal strain distribution showed no differences between various types of joints.

## 4. Conclusions

This study investigated two key structural features of the stem–branch connection in common ash wood and evaluated their mechanical roles using biomimetic 3D-printed T-joints.

The first feature was the ILA located above the knot region. Micro-CT scans confirmed fiber orientations around parenchyma cells, including fibers forming closed, ring-like arrangements. Under mechanical loading (e.g., branch self-weight), this architecture promotes redistribution of shear stresses within the wood. These observations inspired lock-like designs in the 3D-printed T-joints. Bending observations of natural stem–branch junctions suggested that the ILA mainly influences smaller branches. Full-field strain measurements and FEM results showed that ILA improves the redistribution of shear strains within the joint. In mechanical tests of the printed joints, however, ILA did not produce a statistically significant increase in elastic stiffness. ILA reduced deflection at failure by 8%, indicating stiffer behavior in the high-stress plastic regime.

The second feature examined was the fiber orientation in the root–branch and root–crown regions. The asymmetrical 3D-prined model showed a significant 2% improvement in elastic stiffness and 10% lower deflection at failure compared to symmetrical joints. Furthermore, this study highlights an innovative method for plant imaging combined with additive manufacturing technologies to enable functional analysis.

## 5. Patents

The following patents resulted from the work reported in this manuscript.

Lagaňa R., Tončíková Z., Nôta R., Ihring M. (2024). Construction of a stiff joint for use in 3D printing (Konštrukcia pevného spoja na využitie v 3D tlači). Utility Model No. 10299 Y1, filed 9 August 2024; registered 12 March 2025. Slovak Republic: Industrial Property Office. Retrieved from https://wbr.indprop.gov.sk/WebRegistre/UzitkovyVzor/Detail/131-2024?csrt=135437937575 (accessed on 21st November 2025).

Lagaňa, R.; Tončíková, Z.; Nôta, R.; Ihring, M.; Poljak, M. (2025) Construction of a Firm Joint to Be Used in 3D Printing (Konstrukce pevného spoje pro využití ve 3D tisku). Utility Model No. CZ 38784, Industrial Property Office (ÚPV), Prague, Czech Republic, Bulletin No. 37/2025, 2025; 7 pp. Available online: https://isdv.upv.gov.cz/webapp/resdb.print_detail.det?pspis=PUV/42997&plang=CS (accessed on 21st November 2025).

## Figures and Tables

**Table 1:** Stiffness characteristics (N/mm) of stem–branch junction.

Type *	Valid N	Mean	Std. Dev.
c	21	932	417
t	21	989	404
SR_branch_	21	1.018	0.037

* c, compression; t, tension.

**Table 2:** Mechanical properties of 3D-printed joints. Values in brackets stand for a coefficient of variation.

Variable	Type	Valid N	Asymmetrical		Symmetrical	
			no_ILA	ILA	no_ILA	ILA
MOE, MPa	t	10	1658	1674	1657	1611
			(1.7%)	(2.0%)	(1.4%)	(3.3%)
	c	10	1682	1653	1658	1656
			(2.0%)	(1.9%)	(2.1%)	(1.8%)
SR_3D_	t/c		0.99	1.01	1.00	0.97
Strength, MPa	t	10	68.85	70.48	70.80	71.48
			(2.1%)	(1.4%)	(3.6%)	(3.2%)
	c	10	71.41	71.97	71.99	71.79
			(2.9%)	(1.6%)	(2.6%)	(2.2%)
y max, mm	t	10	12.10	11.16	13.08	12.86
			5.6%	7.4%	3.1%	2.7%
	c	10	12.09	12.19	13.16	12.68
			4.8%	3.3%	6.6%	2.1%
y at 16 N, mm	t	10	3.10	3.09	3.17	3.19
			1.5%	1.9%	2.8%	2.2%
	c	10	3.09	3.15	3.21	3.17
			1.1%	1.3%	3.2%	1.0%
y at 16 N, mm	FEM t	1	3.648	3.581	3.564	3.719
	FEM c	1	3.572	3.538	3.564	3.658

## Data Availability

Data are contained within the article or Supplementary Material.

## Supplementary Materials

The following supporting information can be downloaded at: https://www.mdpi.com/article/10.3390/biomimetics11010015/s1, Video S1: Spatial X-ray micro-computed tomography imaging of the ILA above a branch. The cross-section of the upper part of a knot is visible in the mid-bottom part of the section. Video S2: Three-dimensional rotating view of the selected earlywood stem and branch cell lumens in the 2nd annual ring (AR) and the earlywood branch cell lumens in the 1st AR. The color legend of selected earlywood cell lumens: yellow—root-branch lumens of the 1st AR, red—root-branch lumens of the 2nd AR, dark green—right root-crown lumens of the 2nd AR, green—left root-crown lumens of the 2nd AR, light green—root-crown lumens of the 2nd AR twisted around the ILA, blue—ring-like lumens of the 2nd AR in the ILA, pale white—selected two neighboring root-crown lumens of the 2nd AR twisted around the ILA. Video S3: Three-dimensional rotating detail of the ring-like fibers of the ILA in latewood of the 1st AR. The color legend of selected latewood cell lumens: blue—ring-like lumens of the 1st AR in the ILA, red—lumens of parenchyma cells of the rays. The black and white background is the micro-CT section of ring-like ILA.

## Abbreviations

The following abbreviations are used in this manuscript:

ILA	Interlocked area
FEM	Finite element method
T-joint	T-shaped joint of two elements
CT	Computed tomography
micro-CT	Computed tomography at a microscopic level
3D	Three dimensional
PETG	Polyethylene terephthalate glycol
MOE	Modulus of elasticity
